# Parental Psychological Adjustment in Pediatric Acute Lymphoblastic Leukemia: The Mediating Role of Family Functioning and Resilience

**DOI:** 10.3390/cancers17030338

**Published:** 2025-01-21

**Authors:** Ana Ferraz, Susana Faria, Mónica Jerónimo, M. Graça Pereira

**Affiliations:** 1 Psychology Research Centre (CIPsi), School of Psychology, Applied Psychology Department, University of Minho, 4710-057 Braga, Portugal; anasofiaferraz93@gmail.com; 2 Centre of Mathematics (CMAT), Department of Mathematics, University of Minho, 4800-058 Guimarães, Portugal; sfaria@math.uminho.pt; 3 Pediatric Oncology Department, Hospital Pediátrico, Centro Hospitalar e Universitário de Coimbra, 3000-602 Coimbra, Portugal; monica.jeronimo@ulscoimbra.min-saude.pt

**Keywords:** pediatric acute lymphoblastic leukemia, parental distress, psychological well-being, family resilience, family functioning, parental coping, family-centered care

## Abstract

**Background/Objectives:** Acute lymphoblastic leukemia (ALL) is the most common pediatric cancer, with intense treatments affecting both children and their families. Limited information is available on parental distress and psychological well-being during the first treatment year, with most studies focusing on individuals rather than the family system. This study explores longitudinal changes in parental distress (psychological morbidity and traumatic stress symptoms), coping strategies, family resilience and functioning, and psychological well-being. The study also examines the mediating roles of family resilience, family functioning, and coping strategies in the relationships between parental distress and psychological well-being. **Methods**: A prospective study was conducted with 46 parents of children newly diagnosed with ALL, assessing parental distress, family functioning and resilience, coping, and psychological well-being across three treatment phases: consolidation (T0), delayed intensification (T1), and maintenance (T2). **Results**: Parental distress and family resilience significantly decreased from T0 to T2, while parental coping improved over time. Family functioning deteriorated from T0 to T1, stabilizing thereafter. Psychological well-being followed a non-linear trajectory, initially declining from T0 to T1 and improving from T1 to T2. Mediation analyses revealed that family resilience and family functioning partially mediated the relationships between parental distress and psychological well-being. Parental coping did not emerge as a mediator. **Conclusions**: Parental psychological adjustment in the context of ALL is dynamic and influenced by individual and family factors. Interventions that strengthen family functioning and resilience are crucial for supporting parental psychological well-being during treatment. A family-centered approach in healthcare delivery is essential to address individual and systemic challenges.

## Simple Summary

Parents of children with acute lymphoblastic leukemia often face challenges to their psychological well-being due to the nature of the disease and their parental responsibilities. Many experience clinically significant psychological distress, while others seem to adapt. Key factors in this adaptative process include family resilience, family functioning, and parental coping strategies. This longitudinal study aims to explore how psychological well-being, parental distress, coping strategies, and family functioning and resilience change over time. The study also examines the mediator roles of family functioning and resilience and coping strategies in the relationship between parental distress and psychological well-being. The findings highlight that both individual and family factors influence psychological well-being during treatment. Strengthening family resilience and functioning is crucial for supporting parents, and a family-centered approach in healthcare is important in addressing the challenges they face.

## 1. Introduction

Acute lymphoblastic leukemia (ALL) is the most common early childhood type of cancer, characterized as an aggressive form of leukemia marked by an overproduction of lymphocytes in the bone marrow and blood [1]. Over the last two decades, advancements in clinical trials, supportive care [2], and therapeutic approaches have significantly improved outcomes for these children, resulting in higher survival rates [3]. A key factor in this progress is the use of risk stratification, based on patient characteristics, cell biology, and initial treatment response through minimal residual disease (MRD) assessment [4]. In 2019, 14 European countries adopted the ALLTogether protocol, which incorporates MRD and genetic profiling for more precise risk stratification [5,6]. This collaborative protocol, with its multi-phase treatment plan (induction, consolidation 1, consolidation 2, delayed intensification, consolidation 3, and maintenance), tailors treatment intensity according to the group risk (standard risk, intermediate risk, and high risk) [7], optimizing therapy and ultimately improving survival and quality of life (QoL) [8]. Nonetheless, cancer diagnosis remains a source of fear, often leading to major changes in the parents’ lives [2], usually considered life-changing [9]. Thus, the demands of diagnosis and subsequent treatment strategies impact parents’ caregiving roles and well-being [10].

ALL remains a life-threatening condition [11], with parents perceiving a serious threat to their child’s life and the treatment process as complex and invasive [12]. Throughout the treatment process, parents experience frequent hospital visits and admissions, invasive and painful procedures, and health crises, alongside disruptions in their physical, emotional, and social lives due to treatment [13]. The treatment lasts 2 to 3 years, with intensive therapy during the first few months [14], placing family members at greater risk for distress compared to families coping with other cancer diagnoses [15].

As primary caregivers, parents play a crucial role in their children’s adjustment, supporting their coping with illness and treatment while minimizing psychological effects [16]. Their psychological well-being is a key factor for effective parenting intervention [17]. However, studies on parental psychological adjustment and well-being show mixed results, particularly during the first year of treatment, when heightened distress is common [18]. Parenting is often perceived as demanding and stressful, leading to physical and psychological consequences [19] such as symptoms of anxiety, depression [20,21], post-traumatic stress [21,22], distress [23], burnout [24], sleep disturbances [23], and psychosocial and financial burdens [25,26]. Additionally, parents also face significant disruptions in social interactions [27], work-related concerns, and changes in family dynamics [22], further negatively affecting their QoL [28] and psychological well-being [29].

A recent systematic review emphasized the association between parental distress at diagnosis and subsequent adjustment [30], with mental health issues potentially lasting for years after diagnosis [31]. Studies have shown that parents of children with leukemia often report low psychological well-being while caring for their ill child [32,33]. Therefore, these parents may be considered “hidden patients”, requiring special attention to prevent physical and emotional consequences [34]. However, limited descriptive information is available in most studies regarding parental distress throughout the first year of treatment [35,36], underlying the need for longitudinal studies regarding parental distress in childhood ALL [30].

Despite some studies showing that anxiety, depression, and traumatic stress symptoms tend to decrease over time, many caregivers continue to experience clinically significant psychological distress (e.g., [35,36]). Conversely, other studies suggest successful adaptation and coping [37], highlighting the absence of major psychosocial difficulties among parents [38]. Key factors in this adaptative process include family resilience [39,40], family functioning [41,42], and parental coping strategies [33].

Family resilience is a potential resource within the family system [39] that helps parents approach challenges, maintaining stability and avoiding disruptions in family life [43]. Therefore, resilience is crucial for managing cancer-related challenges effectively [44], being directly related to psychological well-being [45]. However, the ability to be resilient changes throughout life and in the presence of a stressor event [46], with some families still struggling with resilience challenges [47]. In this way, longitudinal studies are needed to understand the dynamic changes in family resilience over time and across treatment phases [48].

Family functioning, i.e., the way the family as a whole deals and responds to a child’s illness, impacts the family’s adjustment to a stressor event [41], being positively related to well-being [49]. However, family conflicts are more prevalent in families of children with cancer compared to families of healthy children [50]. As a result, families with poor functioning may be more susceptible to adjustment challenges, as they struggle to manage illness and parenting demands [41]. Research found that family functioning in childhood cancer is negatively impacted, particularly in the first year after diagnosis [50], but resilience can help restore family functioning [39]. Although there is growing awareness of the importance of family functioning in the context of pediatric cancer, most studies focus on individual-level factors, neglecting the family-system perspective [51].

Coping strategies are also central to studies on the psychological well-being of parents caring for children with leukemia (e.g., [33]). Parents adopt several strategies to manage caregiving challenges, which can improve overall well-being [52]. Research has shown that low emotional coping (i.e., less cognitive avoidance, less acceptance or resignation, and less emotional discharge) is linked to better psychological well-being [53], while active coping strategies reduce parental distress a year after the diagnosis [9]. A self-oriented parental coping approach, compared to treatment- or children’s daily care-oriented strategies, uniquely contributed to parents’ psychological well-being [33]. Thus, identifying caregiver coping strategies is essential for developing interventions to improve caregivers’ psychological well-being [54].

The first year of treatment is a particularly vulnerable time for parents [35], making it crucial to understand the trajectory of their psychological well-being, distress (psychological morbidity and traumatic stress symptoms), coping strategies, family functioning, and family resilience. While most studies rely on cross-sectional designs, limited information is available on how families and their members adapt over time [55]. Moreover, few studies have explored parental psychological well-being in this context [9]. Considering the crucial role of the family in childhood cancer [51], more attention should be directed to the impact of family resources on parental adjustment following their child’s diagnosis [42], such as family functioning and family resilience. Furthermore, coping strategies remain insufficiently explored in the context of family adaptation to childhood cancer despite their potential significance [56]. Addressing these gaps could provide valuable insights for the development of family-centered psychological interventions aimed at enhancing the psychological well-being of parents of children with ALL. Studies have emphasized the unique challenges faced by families of children with hematologic cancers (e.g., ALL) and the importance of tailored support resources [57].

This study is grounded on Livneh’s [58] model of psychosocial adaptation to chronic disease, which aims to understand the adaptation process within the context of chronic illnesses. According to this model, adaptation is a dynamic process influenced by various factors, ranging from individual characteristics to family and social contexts. Based on this framework, the present study had the following aims:

1. To assess changes over time in parental psychological morbidity, traumatic stress symptoms, coping strategies, family functioning and resilience, and psychological well-being while controlling for being on leave;
2. To explore the mediator role of family functioning, family resilience, and coping strategies between psychological morbidity and psychological well-being;
3. To explore the mediator role of family functioning, family resilience, and coping strategies between traumatic stress symptoms and psychological well-being.

## 2. Materials and Methods

### 2.1. Study Design

This longitudinal study with three assessment moments is part of a Portuguese multi-centric project addressing the family experience of childhood ALL. The study was conducted at three major Portuguese cancer hospitals, between February 2022 and August 2024. This study received approval from the Ethics Committee for Research in Social and Human Sciences of a major public university (CEICSH 067/2021) and the Ethics Committees of the three hospitals where data collection took place (024/CES; CES.13/022; UIC/1474), and it was performed according to the Declaration of Helsinki. The participants were informed about the study and provided written informed consent.

### 2.2. Participants

A total of 50 parents and their children met the eligibility criteria, and 46 agreed to participate in the present study. Children were eligible if they were diagnosed with ALL for the first time, were six years or younger at the time of diagnosis, and were receiving treatment according to the ALLTogether protocol. Institutionalized children, those with a previous clinical history of oncological disease, and those classified in the high-risk group during the final stratification were excluded. Eligibility criteria for parents were to be the child’s primary caregiver, at least 18 years old, and literate.

### 2.3. Instruments

#### 2.3.1. Sociodemographic and Clinical Questionnaire

This questionnaire was developed for this study to assess parents’ and child’s sociodemographic variables (e.g., sex, age, and marital status) as answered by the participants and child’s clinical variables (e.g., time since diagnosis, duration of hospitalization, and risk group) as answered by healthcare professionals.

#### 2.3.2. Psychological Well-Being Scale (PWS) [59,60]

We utilized a self-report scale that assesses psychological well-being across six dimensions: autonomy, environmental mastery, personal growth, positive relations with others, purpose in life, and self-acceptance. It comprises 18 items rated on a five-point Likert scale ranging from 1 to 5, with higher scores indicating higher levels of psychological well-being. Cronbach’s alphas for the Portuguese version ranged from 0.36 to 0.50. Only the total scale was used in the present study, with a Cronbach’s alpha of 0.84 and McDonald’s omega of 0.82.

#### 2.3.3. Hospital Anxiety and Depression Scale (HADS) [61,62]

This questionnaire evaluates psychological morbidity through 14 items equally divided into two subscales: anxiety and depression. Each item is rated on a four-point Likert scale, ranging from 0 to 3. High scores indicate greater psychological morbidity. In the Portuguese version, Cronbach’s alpha was 0.76 for the anxiety subscale and 0.81 for the depression subscale. In the present study, only the total scale was used, with a Cronbach’s alpha of 0.89 and McDonald’s omega of 0.88.

#### 2.3.4. Impact of Event Scale-Revised (IES-R) [63,64]

This self-report scale evaluates traumatic stress symptoms caused by a traumatic event, across 22 items divided into intrusion, avoidance, and hyperarousal subscales. Participants answered on a five-point Likert scale, ranging from 0 to 4, with higher scores indicating greater traumatic stress symptoms. In the Portuguese version, Cronbach’s alpha ranged from 0.89 to 0.91. Only the total scale was used in this study, with a Cronbach’s alpha and McDonald’s omega of 0.93.

#### 2.3.5. Family Assessment Device–General Functioning (FAD-GF) [65,66]

As a single indicator, the global scale of the FAD evaluates the perception of overall family functioning through twelve items, with six items on healthy and six items on unhealthy family functioning. Scoring is calculated through a four-point Likert scale, ranging from 1 to 4. The higher the score, the more problematic the family’s overall functioning is perceived to be. In the Portuguese version, Cronbach’s alpha was 0.79. In the present study, Cronbach’s alpha and McDonald’s omega were both 0.93.

#### 2.3.6. Family Hardiness Index (FHI) [67,68]

This self-report instrument, consisting of 20 items rated on a four-point scale ranging from 0 to 3, evaluates the internal strengths and durability of the family unit when dealing with stress or adversity through three subscales: commitment, challenge, and control. A higher score indicates greater family resilience. Cronbach’s alpha for the overall scale was 0.76 in the Portuguese version. Cronbach’s alpha and McDonald’s omega, in this study, were 0.88 and 0.87, respectively.

#### 2.3.7. Coping Health Inventory for Parents (CHIP) [69,70]

This instrument assesses coping strategies and styles among parents of children with severe or chronic illnesses, through 44 items. Three coping pattern subscales are included: (I) maintenance of family integration, cooperation, and an optimistic definition of the situation; (II) maintenance of social support, self-esteem, and psychological stability; and (III) understanding the medical situation through communication with other parents and consultation with medical staff. Items are rated on a Likert scale from 0 to 3, with higher scores indicating higher levels of parental coping. In the Portuguese version, Cronbach’s alphas were found to be 0.80, 0.82, and 0.76 for each scale, respectively, and 0.89 for the overall score. In the present study, Cronbach’s alpha and McDonald’s omega for the total scale were 0.87 and 0.88, respectively.

### 2.4. Procedure

**Figure 1:**
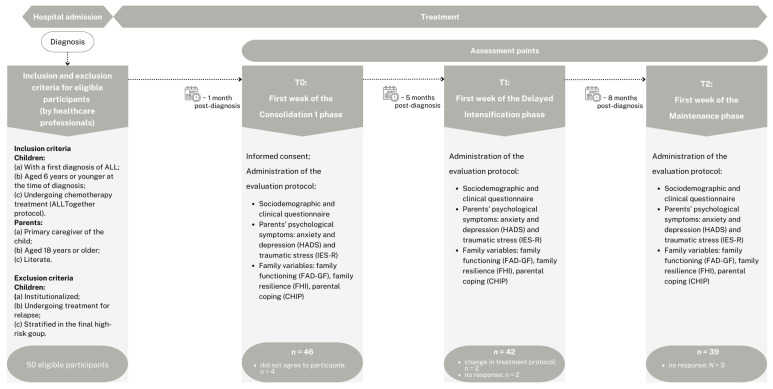
Schematic overview and chronological timeline of the study.

Participants meeting the inclusion criteria were identified by healthcare professionals and informed about the study during the initial phase of treatment. Those who agreed to meet with the researcher in person were subsequently invited to participate in the study. After being informed about the study’s purpose, data confidentiality, voluntary participation, and their right to withdraw without consequences, participants who chose to proceed provided written informed consent. Parents’ psychological morbidity and traumatic stress symptoms, coping strategies, family functioning and resilience, and parental psychological well-being were assessed at three different treatment phases: the first week of consolidation 1 (T0); the first week of delayed intensification (T1); and the first week of maintenance (T2) (see Figure 1).

The first assessment (T0) was conducted in person, and participants answered several questionnaires (i.e., sociodemographic and clinical, anxiety, depressive and traumatic stress symptomatology; family functioning and resilience; and psychological well-being). In the remaining assessment points (T1 and T2), the data were collected through the online software Qualtrics XM, a licensed tool for creating and distributing questionnaires online, which participants were invited to complete individually. For this purpose, participants were contacted and reminded before the data collection moments to ensure the feasibility of all time points. The researcher subsequently sent the link to the questionnaire. The mean time required to complete the self-report questionnaires was 20 min.

### 2.5. Data Analysis

Sample characterization was performed through frequencies and percentages for categorical variables and means and standard deviations for continuous variables, using IBM SPPS Statistics (Statistical Package for the Social Sciences) version 29.

The following statistical analyses were performed using the R Statistical Computing Environment [71]. The courses of parental psychological morbidity (anxiety and depression symptoms), traumatic stress symptoms, family functioning, family resilience, parental coping, and parental psychological well-being over time were modeled using linear mixed models while controlling for parental leave status. The mediation analysis was performed using R and the mediation package [72]. The bootstrapping technique, involving 5.000 samples, was applied to estimate the 95% confidence intervals (CI) for the direct and indirect effects. Indirect effects were considered significant if the 95% CI did not include zero [73].

## 3. Results

### 3.1. Sample Characteristics

The study included 46 parents (39 mothers) at baseline (T0), with a mean age of 35.41 years (SD = 6.77). Most parents lived in urban areas (54.3%) and were married (58.7%) or living with a partner (32.6%). The majority had no higher education (54.3%), and 69.6% of the 42 employed parents were on leave to take care of the children. About 89% of the parents provided more than 18 h of care daily. The children had a mean age of 3.48 years (SD = 1.43), with 63.0% being girls. The subtype of leukemia was predominantly ALL-B (95.7%). On average, children had been hospitalized for 21.96 days (SD = 9.25) and were diagnosed 31.65 days (SD = 5.90) before the assessment.

Participants who dropped out after T0 and T1 did not significantly differ from those who remained in the study regarding their sociodemographic and clinical characteristics. The sociodemographic and clinical characteristics of parents and children at each assessment time point (T0, T1, and T2) are presented in Table 1 and Table 2, respectively.

### 3.2. Differences over Time

**Figure 2:**
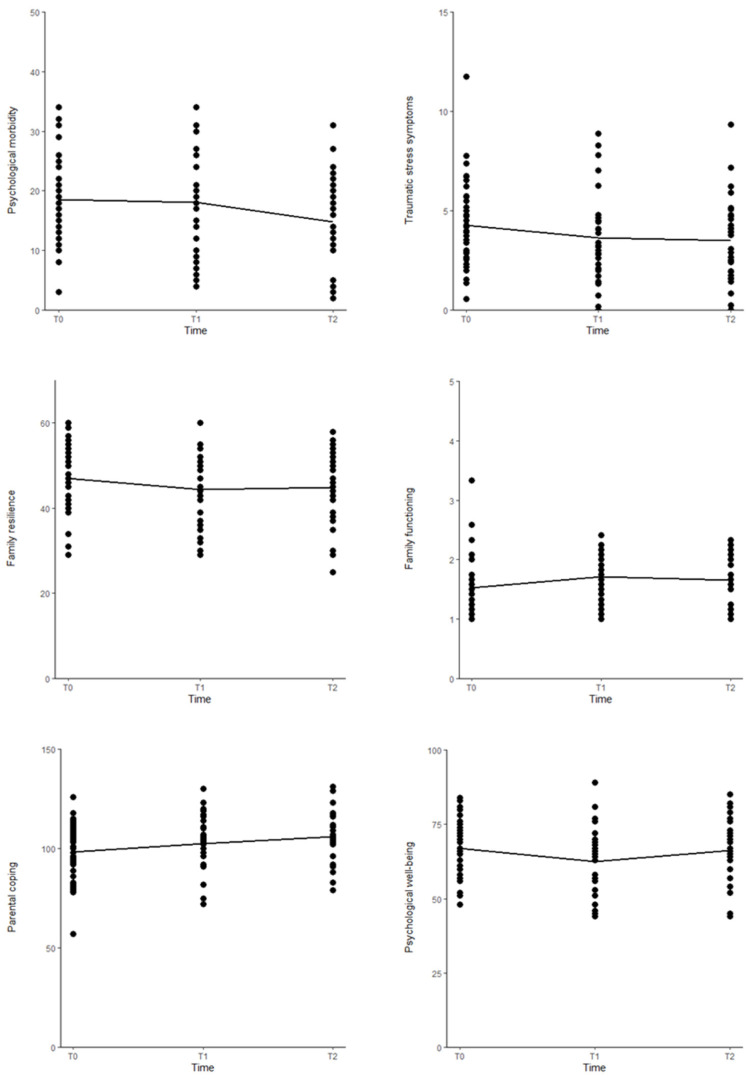
Graphical representation of differences over time.

Parents’ psychological morbidity significantly decreased from T0 to T2 (*β* = −3.73, *p* < 0.01) and from T1 to T2 (*β* =−3.29, *p* < 0.01). Traumatic stress symptoms significantly decreased from T0 to T1 (*β* = −0.66, *p* < 0.05) and from T0 to T2 (*β* = −0.78, *p* < 0.01). Family resilience significantly decreased from T0 to T1 (*β* = −2.58, *p* < 0.05) and from T0 to T2 (*β* = −2.24, *p* < 0.05), and family functioning scores significantly increased from T0 to T1 (*β* = 0.18, *p* < 0.05). Parental coping strategies significantly increased from T0 to T1 (*β* = 4.22, *p* < 0.05), from T0 to T2 (*β* = 7.92, *p* < 0.001), and from T1 to T2 (*β* = 3.07; *p* < 0.05). Psychological well-being significantly decreased from T0 to T1 (*β* = −4.26, *p* < 0.001) and increased from T1 to T2 (*β* = 3.53, *p* < 0.05) (Table 3 and Figure 2).

### 3.3. Mediator Effects

The mediation analysis revealed that family functioning (*β* = −0.02, *p* = 0.005, 95% CI [−0.04, −0.01]) and family resilience (*β* = −0.03, *p* = 0.001, 95% CI [−0.05, −0.01]) partially mediated the relationship between psychological morbidity and psychological well-being. Family functioning (*β* = −0.07, *p* = 0.038, 95% CI [−0.15, 0.00]) and family resilience also partially mediated (*β* = −0.03, *p* < 0.001, 95% CI [−0.05, −0.01]) the relationship between traumatic stress symptoms and psychological well-being. The direct effects remained significant.

Coping strategies did not mediate the relationship between psychological morbidity and psychological well-being (indirect effect: (*β* = −0.01, *p* = 0.120, 95% CI [−0.02, 0.00]) or between traumatic stress symptoms and psychological well-being (indirect effect: *β* = −0.02, *p* = 0.160, 95% CI [−0.07, 0.01]). The direct effects remained significant (Table 4).

## 4. Discussion

Given the longevity and intensity of childhood ALL treatment, the present study aimed to assess changes in parental psychological well-being, psychological morbidity, traumatic stress symptoms, coping strategies, family resilience, and family functioning during the first year following the child’s diagnosis. The study also aimed to investigate the mediating roles of family functioning, family resilience, and parental coping in the relationships between psychological morbidity and psychological well-being and between stress traumatic symptoms and psychological well-being. The findings underscore the importance of understanding parental psychological adjustment in the context of childhood ALL, highlighting the need for integrated healthcare approaches.

Parental distress (psychological morbidity and traumatic stress symptoms) was higher near diagnosis and significantly decreased over time, aligning with previous research [23,30,35]. This decline is consistent with the trajectory of distress reported in early treatment phases, particularly during the intensive induction phase [12,13]. Despite a reduction in psychological morbidity from T1 to T2, no significant decrease was observed from T0 to T1, highlighting the importance of early assessments and interventions during the initial stressful stages of treatment [30]. This is consistent with findings that anxiety and depression symptoms did not decrease over six months post diagnosis [74]. In contrast, traumatic stress symptoms decreased from T0 to T1 and then stabilized, suggesting that parents began adapting to the caregiving role as the acute stress response triggered by the diagnosis decreased [22]. In fact, as the initial stages of medical treatment begin to stabilize [75], the family may start transitioning to its “normal” life, and these traumatic stress symptoms tend to stabilize within the first year post diagnosis [12,36]. These results emphasize the need for ongoing psychological support during the first year post diagnosis and add to the growing body of research advocating for family-centered care to address parental distress throughout treatment [76]. Interventions should be tailored to support parents, immediately after diagnosis, focusing on emotional regulation skills (e.g., cognitive–behavioral strategies, time management, and stress-reduction techniques).

Family resilience showed a decrease from T0 to T1 and T0 from T2, reflecting the significant strain experienced by families in the first months of treatment, leading to a decreased perception of their family’s internal strengths, such as a sense of control, a positive perspective on change, and an active approach to dealing with problems. This outcome is aligned with the idea that early treatment burdens challenge families’ sense of control and optimism. Although some studies offer a clear, stage-based approach to understanding resilience (e.g., [77]), the findings of the present study suggest a more complex and possibly non-linear process, aligning with the view that family resilience is dynamic, complex, and a continuous process, changing with individual family circumstances and the ongoing treatment stress [77].

Family functioning worsened from T0 to T1, confirming the vulnerability of families during the first year post diagnosis [50]. Despite improvements in parental distress, family functioning continued to decline, reinforcing the idea that cancer impacts the entire family system, not just the individual (e.g., [55]). Additionally, since activating the resilience processes may help restore family functioning [39], the decrease in family resilience may help explain the worsening of family functioning. Interestingly, despite the reduction in parental distress over time, family functioning continued to decline, suggesting that while parents may adapt, the broader family system still struggles to maintain balance. This result is consistent with the existing literature, which highlights that cancer affects both individual and family functioning (e.g., [55]), emphasizing the importance of supporting family functioning as part of cancer care [51]. Encouraging regular family meetings or check-ins to assess family functioning and resilience could be one way to address family dynamics, ensuring that parents feel supported in both their individual and collective roles.

Parental coping improved over time, with parents initially devastated by the diagnosis and gradually developing coping strategies [78]. While this increase in coping strategies might initially seem contradictory to the deterioration of family functioning, it reflects the development of coping in response to stress [79]. The initial deterioration of family functioning and resilience may have prompted the development of coping strategies. As these strategies increased, family functioning and resilience stabilized from T1 to T2. Research highlights the positive influence of coping on family functioning and resilience (e.g., [56]). The increase in coping may also explain the reduction in distress, as coping and emotions are dynamically related [80]. A recent systematic review also found a correlation between coping strategies and parental distress [30], emphasizing the importance of promoting coping strategies. Involving parents in support groups, where they can share experiences and strategies, might also be beneficial for improving coping and resilience over time.

Parental psychological well-being showed a fluctuation pattern over time, declining from T0 to T1 and then improving from T1 to T2. This pattern may reflect the interplay between family resilience, functioning, and coping. The initial decline may result from illness and parental responsibilities, increasing caregiver burden and psychological vulnerability [32]. Considering the dimensions of psychological well-being, challenges faced early on may impact parents’ control over their environment, personal growth, autonomy, relationships, purpose in life, and self-acceptance [81]. Also, the observation that 89% of parents provided more than 18 h of care daily highlights both the caregivers’ dedication and the substantial demands of caring for a child with ALL. While this intense caregiving may foster a sense of purpose and deepen the parent–child connection (e.g., [82]), it also poses risks, as prolonged caregiving has been linked to poorer mental health outcomes [83]. This duality underscores the importance of providing comprehensive support systems to help parents navigate the challenges of caregiving while preserving their well-being.

As coping strategies improve and family resilience and functioning stabilize, parental psychological well-being recovers. This improvement from T1 to T2 suggests adaptation to challenges, with research showing that effective coping strategies promote positive well-being [53]. These results emphasize that parental psychological well-being fluctuates, highlighting the need for psychological interventions, especially in the early and intermediate treatment stages.

The study also found that family functioning and family resilience mediated the relationships between parental distress and psychological well-being over time, suggesting that healthy family dynamics buffer the negative impact of distress on well-being. These findings highlight the critical role of family systems in promoting adaptation during crises [41], which is important for parents’ emotional well-being when facing a diagnosis of cancer in the child [42]. A systematic review showed that high-functioning families strengthen internal bonds and access resources, while low-functioning families struggle to cope [84] and are at greater risk for adjustment problems [41]. Supporting family functioning is essential for maintaining psychological well-being [42]. Similarly, family resilience helps parents cope with stressors [43], promoting psychological well-being [44,45]. These findings highlight the importance of interventions targeting family functioning and resilience to improve mental health outcomes. However, while family factors play a significant role, the direct effects of distress on psychological well-being remain substantial, indicating the need for both individual and family-level interventions [85].

Interestingly, parental coping did not mediate the relationship between parental distress and psychological well-being in this study. While coping strategies are usually protective [30], they may not be as effective in this context due to the complex, chronic nature of the stress experienced by parents. Their effectiveness in adjustment may be influenced by other factors of the family’s context (e.g., [86]), such as poor family functioning or low resilience that may diminish their buffering effects. Furthermore, the coping strategies assessed may not fully capture how parents manage their child’s illness, suggesting the need for more specific approaches. The significant direct effects of distress suggest that parents’ immediate emotional reactions may have a greater impact on their psychological outcomes than coping strategies alone. Since coping strategies did not mediate the relationship between distress and well-being, more nuanced approaches that consider the family’s unique context are essential for supporting parents in their caregiving roles.

Despite the notable strengths of this study, such as its longitudinal design, there are limitations, such as the sample size and the absence of a priori power calculation. First, while the sample size warrants cautious interpretation, the representativeness of the data was strengthened by their collection across all hospitals managing ALL, in the country. Additionally, the COVID-19 pandemic also impacted children with oncological conditions, causing delays in surgeries, consultations, and care-seeking behaviors, which likely contributed to diagnostic delays [87]. In Portugal, similar challenges affected healthcare access, either due to patients’ reluctance to seek care or due to the overwhelming pressure on healthcare services; disrupted cancer care and research; and reduced registered cases in the National Oncology Registry in 2020, reversing the previously rising trend in cancer incidence [88]. However, future longitudinal studies should include larger samples. Additionally, the sample mainly comprised mothers (39; 84.9%), although research suggests that mothers are typically the primary caregivers when a child undergoes hospital treatment (e.g., [89]). Another limitation was the use of only self-report measures and the 15% participant loss from T0 to T2. Future studies should include larger samples and aim for a balanced representation of mothers and fathers.

## 5. Conclusions

This study provides valuable insights into the psychological trajectory of parents of children with childhood ALL, emphasizing the importance of early and ongoing psychological interventions throughout the treatment [30]. Distress levels were highest during early treatment phases, with declines in psychological well-being, family functioning, and resilience, highlighting the need for timely psychological support. The findings also underscore the dynamic interplay between individual and family processes, emphasizing the need for interventions that target both levels. Strengthening family dynamics provides a protective foundation, but individual experiences also require special attention. Targeted support for parents is essential to address the psychological distress that persists regardless of family influences. A holistic, family-centered approach is essential, as it reduces psychological distress and improves caregivers’ mental health [76]. Ultimately, the complex, multifactorial nature of parental adjustment highlights the need for integrated healthcare interventions addressing both individual and family factors to improve parents’ psychological well-being during their child’s cancer treatment. Early and ongoing psychological support, along with interventions to strengthen family functioning and resilience, are crucial for families to navigate childhood ALL.

## Figures and Tables

**Table 1:** Parents’ sociodemographic and clinical characteristics at T0, T1, and T2.

		T0 (*n*= 46)	T1 (*n* = 42)	T2 (*n* = 39)
Categorical Variables		*n* (%)	*n* (%)	*n* (%)
Sex				
Male		7 (15.2)	6 (14.3)	5 (12.8)
Female		39 (84.9)	36 (85.7)	34 (87.2)
Residential area				
Urban		25 (54.3)	23 (54.8)	21 (53.8)
Rural		21 (45.7)	19 (45.2)	18 (46.2)
Marital status				
Single		4 (8.7)	4 (9.5)	2 (5.1)
Married		27 (58.7)	24 (57.1)	22 (56.4)
Living with partner		15 (32.6)	14 (33.3)	15 (38.5)
Education				
With higher education		21 (45.7)	19 (45.2)	21 (53.8)
Without higher education		25 (54.3)	23 (54.8)	18 (46.2)
Employment status				
Unemployed		4 (8.7)	5 (11.9)	4 (10.3)
Employed		42 (91.3)	37 (88.1)	35 (89.7)
On leave		32 (69.6)	27 (73.0)	25 (71.4)
Financial impact				
No		30 (65.2)	17 (40.5)	17 (43.6)
Yes		16 (34.8)	25 (59.5)	22 (56.4)
Chronic illness				
No		37 (80.4)	34 (81.0)	32 (82.1)
Yes		9 (19.6)	8 (19.0)	7 (17.9)
Medication				
No		34 (73.9)	32 (76.2)	29 (74.4)
Yes		12 (26.1)	10 (23.8)	10 (25.6)
Daily hours of care				
<6 h		0 (0)	1 (2.4)	1 (2.6)
6–12 h		0 (0)	4 (9.5)	4 (10.3)
12–18 h		5 (10.9)	4 (9.5)	8 (20.5)
>18 h		41 (89.1)	33 (78.6)	26 (66.7)
Presence of other informal caregiver				
No		5 (10.9)	6 (14.3)	8 (20.5)
Yes		41 (89.1)	36 (85.7)	31 (79.5)
Continuous variables	Min–Max	Mean (*SD*)	Mean (*SD*)	Mean (*SD*)
Age	23–52	35.41 (6.77)	35.19 (7.00)	35.56 (6.66)

**Table 2:** Children’s sociodemographic and clinical characteristics at T0, T1, and T2.

	T0 (*n* = 46)	T1 (*n* = 42)	T2 (*n* = 39)
Categorical Variables	*n* (%)	*n* (%)	*n* (%)
Sex			
Boy	17 (37.0)	16 (38.1)	14 (35.9)
Girl	29 (63.0)	26 (61.9)	25 (64.1)
Number of siblings			
0	24 (52.2)	21 (50.0)	20 (51.3)
1	16 (34.8)	16 (38.1)	15 (38.5)
2	6 (13.0)	5 (11.9)	4 (10.3)
ALL subtype			
ALL B	44 (95.7)	40 (95.2)	37 (94.9)
ALL T	2 (4.3)	2 (4.8)	2 (5.1)
Induction type			
Induction A	31 (67.4)	28 (66.7)	29 (74.4)
Induction B	12 (26.1)	12 (28.6)	9 (23.1)
Induction A + C	3 (6.5)	2 (4.8)	1 (2.6)
Risk group			
Standard	NA	12 (28.6)	11 (28.2)
Intermediate low	NA	14 (33.3)	15 (38.5)
Intermediate high	NA	16 (38.1)	13 (33.3)
Clinical complications			
No	13 (28.3)	7 (16.7)	2 (5.1)
Yes	33 (71.7)	35 (83.3)	37 (94.9)
Continuous variables	Mean (*SD*) Min–Max	Mean (*SD*) Min–Max	Mean (*SD*) Min–Max
Age	3.48 (1.43) 1.25–6.00	3.39 (1.37) 1.25–6.00	3.49 (1.46) 1.25–6.00
Number of hospitalizations	1.46 (0.66) 1–3	4.90 (1.75) 7–12	7.18 (2.57) 3–16
Time since diagnosis (in days)	31.65 (5.90) 27–57	145.19 (20) 119–192	256.23 (58.81) 167–373
Hospitalizations duration (in days)	21.96 (9.25) 7–58	35.62 (14.01) 7–84	44.44 (15.31) 22–93

Note: NA = not applicable.

**Table 3:** Regression coefficient estimates of the linear mixed-effects model.

Response Variable	Psychological Morbidity	Traumatic Stress Symptoms	Family Functioning	Family Resilience	Parental Coping	Psychological Well-Being
Fixed effects	*β* (*SE*)	*β* (*SE*)	*β* (*SE*)	*β* (*SE*)	*β* (*SE*)	*β* (*SE*)
Intercept	16.48 (1.74) ***	3.68 (0.46) ***	1.38 (0.10) ***	47.05 (1.78) ***	101.41(3.21) ***	74.06 (1.97) ***
T1	−0.44 (1.04)	−0.66 (0.27) *	0.18 (0.07) *	−2.58 (0.99) *	4.22 (1.95) *	−4.26 (1.09) ***
T2	−3.73 (1.06) **	−0.78 (0.28) **	0.12 (0.07)	−2.24 (1.01) *	7.92 (1.98) ***	−0.73 (1.11)
On leave	2.02 (1.70)	0.60 (0.45)	0.15 (0.10)	0.38 (1.70)	−3.20 (3.14)	−7.20 (1.88) ***

Note. * *p* < 0.05, ** *p* < 0.01, and *** *p* < 0.001; *β* = estimate; *SE* = standard error. On leave: 0 = no, 1 = yes.

**Table 4:** Effects of the mediation model.

Independent Variable → Mediator Variable → Dependent Variable	Effect	Estimate	*p*-Value	95% CI	
				Lower	Upper
Psychological morbidity → Family functioning → Psychological well-being	Indirect effect	−0.02	0.005	−0.04	−0.01
	Direct effect	−0.08	<0.001	−0.12	−0.04
	Total effect	−0.10	<0.001	−0.014	−0.06
Psychological morbidity → Family resilience → Psychological well-being	Indirect effect	−0.03	0.001	−0.05	−0.01
	Direct effect	−0.06	0.003	−0.10	−0.02
	Total effect	−0.09	<0.001	−0.13	−0.05
Psychological morbidity → Parental coping → Psychological well-being	Indirect effect	−0.01	0.120	−0.02	0.00
	Direct effect	−0.10	<0.001	−0.14	−0.06
	Total effect	−0.10	<0.001	−0.14	−0.07
Traumatic stress symptoms → Family functioning → Psychological well-being	Indirect effect	−0.07	0.038	−0.15	0.00
	Direct effect	−0.19	0.010	−0.34	−0.04
	Total effect	−0.26	0.001	−0.42	−0.10
Traumatic stress symptoms → Family resilience → Psychological well-being	Indirect effect	−0.03	<0.001	−0.05	−0.01
	Direct effect	−0.06	<0.001	−0.10	−0.02
	Total effect	−0.09	<0.001	−0.13	−0.05
Traumatic stress symptoms → Parental coping → Psychological well-being	Indirect effect	−0.02	0.160	−0.07	0.01
	Direct effect	−0.24	0.005	−0.39	−0.07
	Total effect	−0.26	0.003	−0.42	0.00

## Data Availability

The data that support the findings of this study are available from the corresponding author [M.G.P.] upon reasonable request.
